# Insect Decline—A Forensic Issue?

**DOI:** 10.3390/insects12040324

**Published:** 2021-04-06

**Authors:** Jens Amendt

**Affiliations:** Institute of Legal Medicine, University Hospital Frankfurt, Goethe-University, Kennedyallee 104, D-60596 Frankfurt am Main, Germany; amendt@em.uni-frankfurt.de; Tel.: +49-69-6301-7571

**Keywords:** forensic entomology, climate change, global warming, development, succession, carrion

## Abstract

**Simple Summary:**

Numerous studies report a decline in insect biodiversity and biomass on a global scale. Since forensic entomology relies on the presence of insects, the question of whether this discipline will be or already is affected by such a decrease is not only posed to investigative authorities and the public, but also to the scientific community. While the data does indeed provide overwhelming evidence of insect decline, even if the methods of evaluation and data pooling are occasionally questioned, only a few studies deal with forensically relevant insects. These few data do hardly prove a decrease in forensically relevant insect species so far. However, one factor driving insect decline is likely to have also a strong influence on necrophagous insects in the future: climate change.

**Abstract:**

Recent reports have shown a dramatic loss in insect species and biomass. Since forensic entomology relies on the presence of insects, the question is whether this decline effects the discipline. The present review confirms that numerous studies document insect population declines or even extinction, despite the fact that the rates of decline and the methods used to demonstrate it are still much debated. However, with regard to a decline in necrophagous insects, there is little or only anecdotal data available. A hypothetical decrease in species diversity and population density in necrophagous insects could lead to a delayed colonization of dead bodies and a modified succession pattern due to the disappearance or new occurrence of species or their altered seasonality. Climate change as one of the drivers of insect decline will probably also have an impact on necrophagous insects and forensic entomology, leading to reduced flight and oviposition activity, modified growth rates and, therefore, an over- or underestimation of a minimum postmortem interval. Global warming with increased temperature and extreme weather requires a better understanding about necrophagous insect responses to environmental variations. Here, transgeneration effects in particular should be analysed in greater depth as this will help to understand rapid adaptation and plasticity in insects of forensic importance.

## 1. Introduction

Nature seems to be losing, as a current United Nations report estimates that more than one million species are at risk of extinction in coming decades [1]. While such statements in recent years have mostly been based on summary studies of the most diverse vertebrate groups (e.g., [2,3]), insects have only become the focus of (public) interest in recent years. Despite the fact that there are more than a million described species of insects (and bearing in mind that another 4.5 to 7 million remain unnamed [4]), time-series data on e.g., ecological aspects and taxonomic nature of insect population trends are rare, compared to vertebrates, and often focused on certain groups of specialized taxa like agriculturally important species [5]. Nevertheless, recent reports have shown a dramatic loss in the biomass of flying insects over a period of less than 30 years [6,7] and sharp declines in the abundance of various insect groups [8,9,10]. Since forensic entomology relies on the presence and functionality of (mostly necrophagous) insects, the question is whether this massive loss of insects calls into question the applicability or flawless performance of this particular discipline. This paper summarizes the current knowledge of this topic and discusses potential issues in forensic entomology.

## 2. What We Know

**Figure 1:**
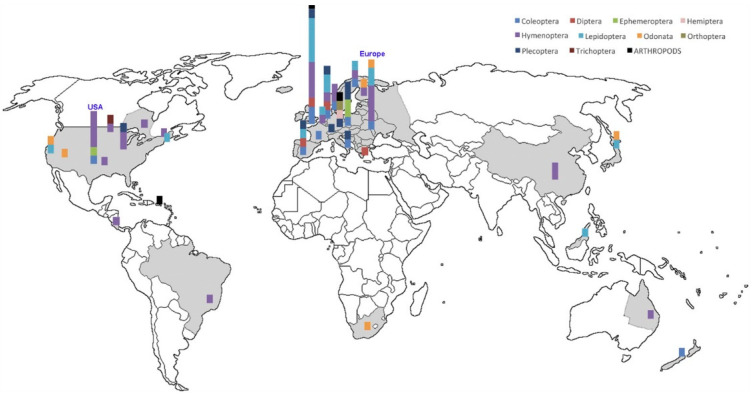
Geographic location of the 73 reports studied on the world map. Columns show the relative proportion of surveys for each taxa as indicated by different colours in the legend. Data for China and Queensland (Australia) refer to managed honey bees only; from [17].

In 2017, [6] measured total insect biomass over 27 years in 63 nature protection areas in Germany and collected a total of 53.54 kg of invertebrates within an average of 176 exposure days per location-year combination, assuming that this amount of biomass represents millions of specimens. They estimated a seasonal decline of 76%, and mid-summer decline of 82% in flying insect biomass over the 27 years of study, regardless of habitat type [6]. This study hit like a bomb and (as of 11 March 2021) has been cited more than 1500 times, triggering a flood of media coverage, conference symposia and special issues on insect decline. Follow-up studies confirmed the drastic decline of terrestrial insects e.g., [11,12,13,14] and led to newspaper headlines such as “The insect apocalypse is here” (The New York Times Magazine, 27 November 2018). However, there are conflicting critical voices about shortcomings in data selection and methodology of such studies like e.g., categorical versus continuous time series, temporal pseudoreplication, the application and comparison of different diversity metrics [15,16] or about data interpretation and communication [17,18]. There are even opposing findings, like the study by Crossley et al. which states an apparent robustness of US arthropod populations and a lack of overall increase or decline [16]. However, the majority of studies confirm a decrease and just disagree about its magnitude, and [5] recently summarises that, despite much variation across time, space, and taxonomic lineage, reported rates of annual decline in abundance frequently fall around 1% to 2%, which is a worrying number. It is pointed out by [14] that most of the studies on insect decline are restricted geographically and taxonomically. While the former can be easily verified by looking at the exemplary map of the world from the study by Sánchez-Bayo and Wyckhuys ([17] Figure 1), using total insect biomass as a proxy for biodiversity [6] or aggregating data across higher taxonomic categories and ecological groups (e.g., [16]) might help to deal with the complexity of population-level stochasticity in insects, but often overlook species-level trends [5].

It must be stated once again that the study that fuelled the whole current discussion [6] does not offer any taxonomic resolution, but merely caught and weighed “flying insects”. Taxon-specific findings could hardly be derived here. Nevertheless, we can refer to some works to better assess the extent of the situation at order or family level. The majority of studies on terrestrial insects deal with the orders Coleoptera, Hemiptera, Hymenoptera, and Lepidoptera [18]. Hallmann et al. ([19]) found in a 30 year survey in the Netherlands that ground beetles (Coleoptera: Carabidae) showed a mean annual decline of 4.3% in total numbers over the period of 1985–2016 and calculate a reduction in total biomass of at least 42% for ground beetles. Interestingly, they showed an increase in carrion-beetles (Silphidae) and explained that by carrion experiments done close to the traps. This illustrates how highly context- and taxon-specific temporal changes in insect populations must be evaluated. Bell et al. [20] showed that aphid annual totals fluctuated widely in the UK, but this group was in a steady state over the long-term, with a non-significant decline of −7.6%. Such a trend may have been driven by three of the most abundant species, highlighting the need to work and understand the taxonomy of the target taxa.

Carvalheiro et al. [21] compared in three European countries four 20-year periods of rapid land-use intensification and natural habitat loss (1930–1990) with a period of increased conservation investment (post-1990) and found extensive species richness loss for bumblebees before 1990. In the same study authors found that richness of butterflies in the UK, Netherlands and Belgium declined from 1950 to 2009, and [22] showed for the tropical island state of Singapore that 32% of 413 recorded species of butterflies eradicated since 1854.

**Figure 2:**
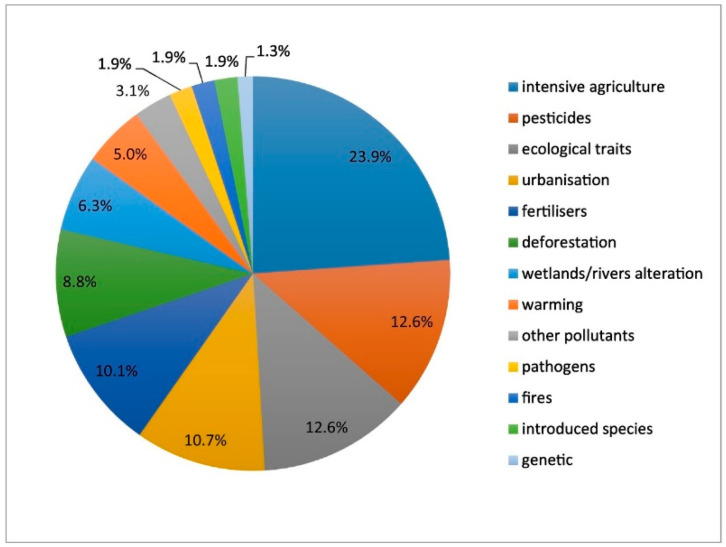
Main factors associated with insect declines according to reports in the literature (from [17]).

These examples confirm the described trend of decline for selected groups [18]. The main causes of the aforementioned insect decline are rapid urbanisation and habitat homogenisation, industrialisation and agricultural expansion based on monocultures, and the use of pesticides like neonicotinoids. Reference [17] identify agriculture associated factors and pesticides as the main drivers in over 1/3 of the studies on insect decline (Figure 2).

Once you have gained an overview from the literature, you will quickly realise that there are little or no data available for the families that govern forensic entomology. Here, taxa from the order Diptera and Coleoptera dominate carrion globally and thus also human cadavers [23,24,25,26,27,28]. Studies on the decline of Coleoptera mainly deals with Carabidae, saproxylic beetles, or aquatic taxa, or highly specialised species like fireflies. When it comes to carrion associated taxa, evidence for decline is rare. References [29,30] discuss the decline of some groups of dung beetles, which play an important role especially in the carrion community of tropical habitats, and [31] found in Italy a relative decrease in frequency of roller species (an ecological subgroup of dung beetles) by 31%. Even though Staphylinidae can be present with quite a large diversity of species on carrion and often occur there as predators, data are available mainly on other ecological guilds of this family, e.g., saproxylic taxa [32]. The already mentioned increase in the number of Silphidae specimens [19] should be cited with caution due to its possible bias caused by bait and carrion experiments nearby the sampling area. However, also [33] confirm a steady state or even increase of Silphidae during two periods of sampling in New Hampshire/USA in 1973–1977 and again in 2015–2017. The state of knowledge is not much better for the most important group in forensic entomology, the Diptera. Most of the insect decline studies focus on hoverflies, which are of particular ecological and system-maintaining importance due to their role as beneficial insects in pest control and pollination [21,34,35]. Despite the fact that such approaches neglect or even ignore the importance of non-syrphid flies as pollinators [36], they lead to a surprising knowledge gap when it comes to the decline of necrophagous Diptera. The most important families due to their abundance on human bodies and often timely occurrence post mortem are the Calliphoridae, Sarcophagidae and Muscidae. While there are numerous data on their presence, seasonal occurrence and succession on carrion, there are almost no long-term studies. The only exception are studies on climatic change and warming in the Arctic. Here, the Muscidae are in focus, as they play a key role in these extreme habitats as pollinators [37]. Reference [38] showed a significant decline of Muscidae from 1996 until 2009 for high-Arctic Greenland and [39,40] confirmed such significant declines in 7 of the 14 muscid species found in five or more years between 1996 and 2014, as well as a dramatic (80%) decrease in diversity and abundance in some habitats. Beyond that, there are only avenues to speculate on potential trends. Amendt (unpublished data, [41]) and [42,43,44] sampled necrophagous insects in Frankfurt (Germany) and the surrounding area over a period of 20 years and could not detect any change in species diversity; moreover, the number of insect-infested corpses did not decline, on the contrary, one rather gets the impression (i.e., not statistically proven) of an increase if one looks at the number of insect-infested corpses at the Institute for Legal Medicine in Frankfurt. However, as the respective research question of these studies determined a diversity of different methods (trap and bait type, collection intervals, species selection, etc.) these data can only be used with caution for a serious evaluation of necrophagous insects decline.

In summary, there are numerous well-documented examples of insect population declines and extinctions in the context of the anthropogenic drivers of global change, and even the rate of those declines is still hotly debated, fuelled by a lack of standardised, systematically collected data. With regard to a decline in necrophagous insects or possibly opposing trends, there are little or only anecdotal data available.

## 3. What We Do Not Know (But Could Be…)

Even though there are currently no reliable data on a decline in forensically relevant insects, it makes sense to address the issue. After all, it cannot be ruled out that the factors that are driving back numerous insect species and populations have at least some influence on the biological and ecological characteristics of necrophagous insects and thus ultimately on the informative value of forensic entomology.

Before discussing possible impacts of insect decline on necrophagous invertebrates, there is one major issue to emphasise: reduced resource availability, e.g., due to urbanisation or agriculture, is a very important aspect when understanding insect decline. Flower visitor declines, for example, have been linked to changes in resource availability [45]. But resource availability has always been a challenge for necrophagous insects, since carrion is an unpredictable, patchy and ephemeral resource (whether a dead mouse or a human body), and its exploitation and use as feeding and breeding habitat poses particular challenges to its necrophagous community on a spatial and temporal scale. Quantitative data on carrion biomass are lacking e.g., due to methodological issues [46,47,48] and it is difficult to evaluate whether carrion availability is (or will be) reduced if there is no baseline to refer to. It seems reasonable, however, not to assume any reduction; there is, for example, a clear increase in rodent populations in the US (which is thought to be linked to a rise in temperature, among other things)—and an increase in living animals will sooner or later also lead to an increased supply of carrion. Therefore, typical drivers of insect decline will probably not lead to a reduced resource availability for necrophagous insects. However, there could be a change in the composition of the carrion supply, away from large vertebrates (e.g., because of loss or modification of natural habitats) towards small species such as rodents, where at least certain pests could be the winners of climate change. This in turn would possibly have an influence on the species composition of the necrophagous community since, for example, not all fly species colonise small carcasses. In Europe, large vertebrate carrion, including human cadavers, can attract many species of Sarcophagidae (Diptera: Flesh flies), but only a few of them utilize it for their offspring, i.e., larvi- or oviposit on it [49]. Additionally, in various baiting experiments with small rodent carcasses, the author always finds the flesh fly *Sarcophaga caerulescens* (Zetterstedt, 1838) but never *S. argyrostoma* (Robineau-Desvoidy, 1830), the only flesh fly species of forensic relevance in Central Europe. The situation is similar for the blow fly *Protophormia terraenovae* (Robineau-Desvoidy, 1830)—this species is of certain forensic relevance due to its common presence on human bodies, but seems to ignore small carrion. Hence, a shift towards more small carrion might favour certain taxa and modify, therefore, the species composition and abundance. However, a reduced species diversity would not necessarily have to be a disadvantage from a forensic-entomological point of view: 4–5 species might be easier to deal with than 24–25 species. At the end of the day, it could even be an advantage to work with a small number of species, since one would have to study and know a smaller set of species in terms of their biology and ecology Less reference data on growth and less ecological data on phenology need to be gathered, identification becomes easier due to fewer possibilities for confusion and error, and even the appropriate sampling and further rearing of insect evidence could be easier. However, in the worst case, species relevant for the assessment of the succession interval become rarer or will even disappear, and thus the templates and patterns defined on the basis of previous studies can no longer be applied in certain cases or simply lead to less accurate estimates. A decline in species leading to only one fly species instead of three or four species developing on the corpse after e.g., a postmortem interval (PMI) of 8 days could impact the estimation of an accurate date based on the age of those specimens for the same reason, as data based on e.g., three or four different species and their development rates provide more certainty in the assessment. However, one should not lose sight of the fact that there is already now no high species diversity on one and the same body, especially during the first days or even weeks postmortem [43]. Reference [42], for example, found 13 species of flesh flies attracted to baited traps, but on human corpses just one of them, *S. argyrostoma*, is representing the family in central Europe almost exclusively. Hence, a small number of species on dead bodies does not necessarily have a negative effect when it comes to PMI estimation as forensic entomology is already used too.

Reduced species diversity and insect abundance could lead to delayed colonisation, but it is not proven so far that a smaller number of species means a lower number of colonised corpses and a higher number of insect-free bodies. Instead, the low number of species which succeed the competition could be much more present than before. Typical “winners” could be highly adaptive and competitive native species such as *Lucilia sericata* (Meigen, 1826), or invasive taxa like several species of the genus *Chrysomya* (Robineau-Desvoidy, 1830, e.g., *Ch. albiceps* (Wiedemann, 1819) or *Ch. megacephala* (Fabricius, 1784).

**Figure 3:**
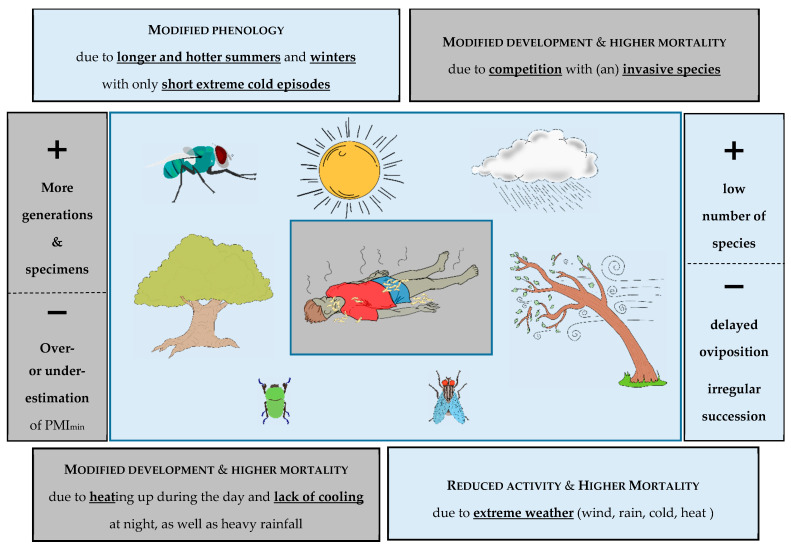
Global warming as a driver of insect decline and its possible impact (**+** and **−**) on the necrophagous fauna and forensic entomology (grey background: juvenile stages, light blue: adult stages).

One driving factor of insect decline is climate change, and global warming and extreme weather (e.g., heavy rain, storms and droughts) are one of its most important effects. It can be expected that they will also have an impact on necrophagous insects, whereby one must distinguish between two targets: The “adult level” and the “juvenile level” (Figure 3).

Increased (average) temperature and frequency of extreme weather events will impact the activity and behaviour of adult insects, but the direction of such an effect is not clear. Heavy wind or rain might decrease the rate of detection of carrion due to reduced flight activity and the scattering of odours that are relevant for cadaver discovery. Due to milder winters and longer summers, adult activity will start earlier and end later over the course of the year. Low temperatures are generally a key factor limiting the range of insect species, and even a small increase in winter temperatures enables survival in areas that were previously inaccessible. The adult phenology of single species will, therefore, change. Beside this shift (or extension) of activity, species will be able to access more habitats, e.g., be found in higher altitudes as insects move uphill to escape warming temperatures. All this could increase the timely detection and colonization of dead bodies in more habitats and for a longer period of the year. A shift in temperature profiles could enable larger range expansion due to higher chances of winter survival, as it is typical for invasive species like *Ch. albiceps* [50], *Ch. megacephala* [51] or *L. cuprina* (Wiedemann, 1830) [52]. The more common presence could affect the local fauna due to interspecific interactions and competition and there might be negative effects of invasive species (see also above “What we know”). But even though this is regrettable for many reasons, it is not proven whether there will be a negative effect for routine work in forensic entomology. Of course, a lack of awareness about the possible presence of invasive species in the native region could lead to misidentifications and incorrect age determinations [52]. Moreover, there is no doubt that certain biological characteristics like the predatory behaviour of the larvae of that invasive species have a negative impact on the presence, abundance and maybe even development of native species. But I also put forward the hypothesis that a small number of species (the “winners”) might be more to hand and useful for the expert than several dozens of different taxa. Nevertheless, a low number of species could lead to a decreased population and, due to e.g., hot weather and high wind speeds, climate change might lead to a reduced flight and oviposition activity and as a consequence to a delayed colonization of dead bodies. Moreover, existing succession templates may need to be revalidated or adapted.

The juvenile stages on a dead body in the field could experience increased mortality or possibly aberrant growth behaviour due to periods of extreme heat or heavy rainfall. Temperatures can also impact the transition to the next developmental stage in summer. The effects of mild winter temperatures and extreme summer temperature fluctuations on the development of the local necrophagous fauna have hardly been investigated so far. New invasive taxa could eliminate native species through cannibalism and cause altered growth through competitive situations. Such alteration or modification might lead to over- or underestimations of a PMI_min_ and, therefore, impact the outcome of the entomological report. An exciting interface between the adult and the juvenile stages are the so-called transgenerational effects. Phenotypic plasticity across generations is an important mechanism for organisms to cope with climate variability at different temporal scales [53]. In the context of fast climate change and increase in temperature fluctuation and unpredictability, a better knowledge about response to environmental variations through such transgeneration effect may help to understand the ability of rapid adaptation and plasticity [53,54]—an issue which so far has been neglected in forensic entomology.

## 4. Conclusions

Up until now there has been no evidence for a decline in necrophagous insects. If there is any indication, it has no relevance for forensic entomology so far. This need not always be the case, and it is quite possible that single drivers of insect decline will sooner or later also affect forensically important taxa. Here, in particular, climate change and global warming can be named. It cannot really be expected that these factors will result in a decline, but they will have a strong impact on the biology and ecology of necrophagous insects. In this respect, therefore, it is not the decline in insects but climate change that will have a strong effect on forensic entomology and which research will have to address in the future. Here, the focus should be on transgenerational plasticity, because climate change will pose major challenges to the short-term adaptive abilities of insects. Existing associations such as the European Association for Forensic Entomology or the North American Forensic Entomology Association should establish a network of baseline data on the occurrence and activity of necrophagous insects, backed up with the relevant local climatic data and next-generation effects. Such cooperation and interaction will help to better understand key species and to better target the impact of climate change on forensic entomology, for example, by identifying specific factors such as e.g., droughts or the presence of heat islands in urban environments, as key players in triggering transgenerational effects and associated modified development.

## Data Availability

Not applicable.
